# The effect of compositional fluctuations in a liquid Fe–O alloy on the nucleation of Earth’s inner core

**DOI:** 10.1038/s41598-025-07258-1

**Published:** 2025-07-01

**Authors:** Alfred J. Wilson, Monica Pozzo, Christopher J. Davies, Andrew M. Walker, Dario Alfè

**Affiliations:** 1 School of Earth and Environment, University of Leeds, Leeds, LS2 9JT UK; 2 Faculty of Technological and Innovation Sciences, Universitas Mercatorum, 00186 Rome, Italy; 3 Department of Earth Sciences, University College London, London, WC1E 6BT UK; 4 Institute for Materials Discovery, UCL East, London, E20 2A UK; 5 London Centre for Nanotechnology, Thomas Young Centre, University College London, London, UK; 6 Department of Earth Sciences, University of Oxford, Oxford, OX1 2JD UK; 7 London Centre for Nanotechnology, University College London, London, WC1H 0AH UK; 8 Dipartimento di Fisica “Ettore Pancini”, Università di Napoli Federico II, 80126 Naples, Italy

**Keywords:** Core processes, Mineralogy, Core processes, Mineralogy, Core processes, Mineralogy

## Abstract

The Earth’s solid inner core plays a fundamental role in determining the past and present properties and dynamics of the Earth’s deep interior. Inner core growth powers the geodynamo, producing the protective global magnetic field, and provides a record of core evolution spanning geological timescales. However, the origins of the inner core remain enigmatic. Traditional core evolution models assume that the inner core formed when the core first cooled to its melting temperature, but this neglects the physical requirement that liquids must be supercooled to below their melting point before freezing. Prior estimates from mineral physics calculations of the supercooling $\delta T$ required to homogeneously nucleate the inner core from candidate binary alloys exceed constraints of $\delta T \lesssim 400$ K inferred from geophysical observations, while a plausible scenario for heterogeneous nucleation has yet to be identified. Here we consider a different possibility, that atomic-scale compositional fluctuations can increase the local melting temperature, and hence supercooling, available for homogeneous nucleation. Using molecular dynamic simulations of Fe-O alloys we find that compositional fluctuations producing O-depleted regions are too rare to aid nucleation, while O-enriched regions can reduce the undercooling by $\sim$50 K ($\delta T \sim 700$ K) for a bulk concentration of 20 mol.% O or $\sim$400 K ($\delta T \sim 300$ K) for a bulk concentration of 30 mol.% O. While these results do not explain the nucleation of Earth’s inner core, they do show that compositional fluctuations can aid the process of homogeneous nucleation.

## Introduction

The solid inner core plays a key role in the deep Earth system. Growth of the inner core is thought to be responsible for the global magnetic field which shields Earth’s surface from harmful solar radiation. As the Earth slowly cools the inner core freezes outwards, releasing latent heat and light elements which provide the main source of convective buoyancy driving generation of the global magnetic field in the overlying liquid outer core^1,2^. The presence of the inner core divides the liquid core into distinct dynamical regions^3^ and has been linked to the existence of stable layers above the inner core boundary^4^ (ICB) and below the core-mantle boundary^5–7^, which influence convection and magnetic field generation. Before inner core formation the core was above its melting point everywhere and the dynamo process was likely less efficient than the present-day^2,8^. The event of formation may therefore have produced distinct magnetic field behaviour^9,10^ that can be observed by paleomagnetism^11,12^. The process and timing of inner core formation is therefore essential for understanding the thermal evolution of the deep Earth, including its present state, and the interpretation of the palaeomagnetic record^13,14^. However, at present, the origins of the Earth’s inner core remain unclear.

Traditional models of core evolution^2,8,15^ assume that the inner core nucleated when the temperature of the core, *T*, fell to the melting temperature $T_{\textrm{m}}$ at the centre of the Earth. However, this ignores the physical requirement that liquids must be supercooled by an amount $\delta T= T_{\textrm{m}}- T$ below the melting point before solids can nucleate from them^16^. Crucially, the interface that must be established between the first solids and residual liquids comes with an energetic penalty, which inhibits further growth and can only be overcome through the stochastic nature of freezing at the atomic scale. This inhibition is diminished with greater supercooling, meaning a liquid will spontaneously freeze sooner if supercooled to a greater degree. While the value of $\delta T$ is poorly constrained^17^, mineral physics calculations have shown that 500–1000 K of supercooling is required to nucleate the inner core from pure Fe^18–21^ or simple binary compositions^19,22^. By contrast, the maximum available supercooling estimated from geophysical observations of the core’s thermal structure is 420 K^22^ and a more complete consideration of geophysical constraints requires^17^ $\delta T< 100$ K. Alternative nucleation mechanisms including heterogeneous nucleation, density fluctuations and radiogenic processes have not been found to be viable for inner core formation^17–19^. It is therefore unclear how an inner core of the presently observed size was able to form.

Previous studies of inner core nucleation have focused on one and two component systems^18–22^, specifically pure Fe and those with light elements which are thought to be present in the core (O, C, Si, S) due to cosmochemical abundances^23^ and the density structure observed by seismology^24^. These studies find that the supercooling required for homogeneous nucleation of the inner core is always incompatible with geophysical constraints (see ref^17^ for a review). Considerations of heterogenous nucleation have failed to identify a viable pre-existing solid surface which could be present in the liquid core to initiate nucleation^17,18^. Other effects not usually considered within the classical paradigms of homogeneous and heterogeneous nucleation, such as pressure perturbations and radioactivity, have also been investigated and are unlikely to substantially reduce the nucleation barrier^17–19^. Here we investigate a different effect, based on compositional fluctuations in supercooled Fe-O liquids.

Oxygen is an important candidate light element in Earth’s core because it partitions strongly into the liquid phase on freezing, which can explain the observed density contrast at the ICB. This partitioning results in a depression of the melting temperature, $\Delta T_{\textrm{m}}$, which for likely core oxygen concentrations^25^ of $c \approx 10-20$ mol.%, is well approximated by the linear formula $\Delta T_{\textrm{m}}\approx T_{\textrm{m}}^{\textrm{Fe}}(1 - c/s_{Fe})$, where $T_{\textrm{m}}^{\textrm{Fe}}$ is the melting temperature of pure iron and $s_{Fe}$ is the entropy of melting of pure iron^19^. Depending on the exact value of *c*, $\Delta T_{\textrm{m}}\approx 600-1000$ K.

Statistical fluctuations cause the oxygen concentration at the atomic scale to deviate from the mean uniform value of the macroscopic mixture, and can in principle produce regions that are almost pure Fe. If the mixture is supercooled by an amount $\delta T^{\textrm{mix}}= T - T_{\textrm{m}}^{\textrm{mix}}$, for example 300 K, below its melting temperature $T_{\textrm{m}}^{\textrm{mix}}$ then these regions are supercooled by an amount $\delta T^{\textrm{Fe}}= T - T_{\textrm{m}}^{\textrm{Fe}}\approx 900-1300$ K below their own local melting temperature $T_{\textrm{m}}^{\textrm{Fe}}$, and this supercooling may exceed, even substantially, the estimated amount needed to homogeneously freeze the core. However, this benefit is offset by the reduced probability of fluctuations producing a highly O-depleted region, compared to the situation where nucleation is equally probable in the whole supercooled region. Analogous arguments apply to O-enriched regions, assuming that the melting temperature of FeO is comparable to that of pure Fe^26^ and greater than the mixture. Our previous studies^19^ on liquid Fe-O alloys likely did not observe nucleation events due to compositional fluctuations due to their rarity in such small volume and short duration simulations.

In this study we use new large volume (64,000 atoms) and long duration (> 200 ns) molecular dynamic simulations to search for supercooled regions driven by compositional fluctuations in Fe-O alloys at core pressure-temperature conditions. Compositions satisfying the core’s seismologically inferred elastic properties commonly favour high oxygen concentrations^27^, therefore we focus of Fe-O alloys because fluctuations in these systems can produce local differences in compositions and $T_m$ of 10s of percent and hundreds of degrees. We calculate the occurrence probability of O-depleted and O-enriched regions and use this information to estimate the waiting time required to observe nucleation events, which can be used to constrain viable scenarios for inner core formation.

## Compositional fluctuations in supercooled liquid alloys

We use Classical Nucleation Theory^16^ (CNT) as a theoretical framework for quantitatively analysing compositional fluctuations in supercooled liquids and evaluating the average time a system will take to freeze for a given $\delta T$. Molecular dynamic studies of nucleation at core conditions have revealed non-classical behaviour, most importantly the formation of metastable phases not predicted by CNT^20,21,28^. Whilst the framework of CNT does not include these mechanisms or other complexities such as non-Arrhenius behaviour, it has proven useful in describing the nucleation rates of previous studies^19–22,28^. We therefore apply the same theory here, in the absence of a more general toolkit. CNT defines the rate *I*(*r*) at which a solid nucleus of size *r* forms within a supercooled system as

1 $$\begin{aligned} I(r) = I_0 \exp {\left( -\frac{\Delta G}{k_B T}\right) } \end{aligned}$$

where $I_0$ is a kinetic prefactor, scaling the nucleation rate of a specific system, $k_B$ is the Boltzmann constant, and $\Delta G$ is the total free energy change defined as

2 $$\begin{aligned} \Delta G = \frac{4}{3} \pi r^3 g^{sl} + 4 \pi r^2 \gamma . \end{aligned}$$

Here, $\Delta G$ is assumed to capture all components of the energetic penalty of forming a nuclei and is the sum of contributions from $g^{sl}$, the free energy change per unit volume associated with converting liquid to solid, and $\gamma$, the free energy per unit area associated with establishing an interface between solid and liquid. $g^{sl}$ varies with $\delta T$ as

3 $$\begin{aligned} g^{sl} = h_f \frac{\delta T}{T_{\textrm{m}}}h_c\left( \delta T\right) \end{aligned}$$

where $h_f$ is the enthalpy of freezing, and $h_c$ is a corrective term to account for non-linearity in the dependence of $g^{sl}$ on $\delta T$. This means that, by definition, *I* evolves exponentially with $\delta T$ and that at the same temperature, two systems of different composition can have nucleation rates differing by many orders of magnitude.

When nuclei are small, the energetic penalty of forming an interface with the surrounding liquid outweighs the energetic benefit of converting liquid to solid. Therefore, while small nuclei do form spontaneously, growth is less likely to occur than re-melting until a critical size is reached, after which further growth is energetically preferred to re-melting. This critical size $r_c$ is located at the maximum of $\Delta G (r)$ and is given by

4 $$\begin{aligned} r_c = -\frac{2 \gamma }{g^{sl}}. \end{aligned}$$

When nuclei larger than $r_c$ form, they are likely to cause spontaneous freezing of the entire system.

The average waiting time $\tau _w$ before a critical nucleus will form is the inverse of $\frac{1}{2} I(r_c)$ (because only half the nuclei of radius $r_c$ will avoid re-melting), given by

5 $$\begin{aligned} \tau _w = \frac{\tau _0}{V_{IC}} \exp {\left[ \frac{16}{3}\frac{\pi \gamma ^3 T_{\textrm{m}}^2}{k_B T \delta T^2 h_f^2 \left[ h_c\left( \delta T\right) \right] ^2}\right] } \left( \frac{1}{P} \right) = \frac{\tau }{P}. \end{aligned}$$

Here $\tau _0$ is a system specific pre-factor which can be evaluated from molecular dynamic simulations (see Wilson et al. for details^22^), $V_{IC}$ is the volume where $\delta T< 0$, which at a maximum is equal to the inner core volume, $P(n,\delta T)$ is the fraction of the system that contains compositional fluctuations large enough to host a critical nucleation event associated with an undercooling of $\delta T$ and $\tau$ is the waiting time for the composition of a fluctuation but with a volume equal to $V_{IC}$. For conventional homogeneous nucleation without compositional fluctuations, the system has a single composition and *P* = 1. When the composition can fluctuate locally and nucleation is expected to occur within the fluctuations $P < 1$.

If the liquid core behaved as a perfect gas, then *P* would follow a Poisson distribution with average and variance equal to *N*. For *N* large enough the central part of the Poisson distribution is well approximated by a Normal distribution, with average and variance also equal to *N*. We therefore write *P* as

6 $$\begin{aligned} P(n,\delta T) = \exp {\left[ -\frac{1}{2}\left( \frac{n - N}{\sqrt{N \sigma }} \right) ^2\right] } \frac{1}{\sqrt{2 \pi N \sigma }}, \quad \text{ where } \quad N(\delta T) = 1.41\times 10^{29}. \frac{4}{3}\pi r_c(\delta T)^3 c, \end{aligned}$$

where *n* is the number of O atoms observed in a sub-volume of the simulation and $\sigma$ defines the curvature of the distribution. *N* is the average number of O atoms per sub-volume, where *c* is the bulk oxygen concentration, $v_c = 4 \pi r_c^3/3$ is the volume of the region based on the required critical radius, and the prefactor gives the number of iron atoms in this volume based on the core density^29^ of 13090 kg $\hbox {m}^{-3}$. The relevant size of this sub-volume is based on the critical radius of the mixture, because at this size the solid embryo is equally likely to grow as it is to remelt. This critical size is therefore based on the actual supercooling of the mixture, $\delta T^{\textrm{mix}}= T - T_{\textrm{m}}^{\textrm{mix}}$. The probability associated with regions of pure Fe and pure FeO are given by $P(0,\delta T^{\textrm{mix}})$ and $P(N/2,\delta T^{\textrm{mix}})$ respectively.

The role of compositional fluctuations in the nucleation process can be established from Eq. (5). In the absence of compositional fluctuations, $T_m$ is the melting temperature of the mixture $T_{\textrm{m}}^{\textrm{mix}}$ and $P=1$. Regions that are depleted in O will have a locally higher melting temperature and larger $\delta T$ with the net effect being to reduce $\tau$ and therefore $\tau _w$, but these regions only arise in a small fraction of the system ($P<1$), which increases $\tau _w$. The balance between increased nucleation rates caused by compositional fluctuations and the rarity of such fluctuations is therefore key for assessing whether this mechanism can reduce the supercooling required to nucleate the inner core. The key is that in nucleating a pure iron region, the relevant supercooling temperature entering the $\tau$ term in Eq. (5) is the one referred to pure iron, which is therefore increased by the difference $T_{\textrm{m}}^{\textrm{Fe}}- T_{\textrm{m}}^{\textrm{mix}}$ (i.e. by 600 K for the 10% mixture) compared to the supercooling temperature of the mixture, but the size of the critical radius is the one that would allow the mixture to fully freeze, which therefore has to be evaluated at the mixture supercooling temperature $\delta T^{\textrm{mix}}$. Mathematically, Eq. (5) becomes

7 $$\begin{aligned} \tau _w = \frac{\tau (\delta T^{\textrm{Fe}})}{P(\delta T^{\textrm{mix}})}. \end{aligned}$$

Analogous arguments apply to O-enriched regions, assuming that the melting temperature of FeO is comparable to that of pure Fe^26^. In this case $\delta T^{\textrm{Fe}}$ in Eq. (7) is replaced by the supercooling compared to pure FeO, $\delta T^{\textrm{FeO}}= T - T_{\textrm{m}}^{\textrm{FeO}}$.

## Results

**Fig. 1:**
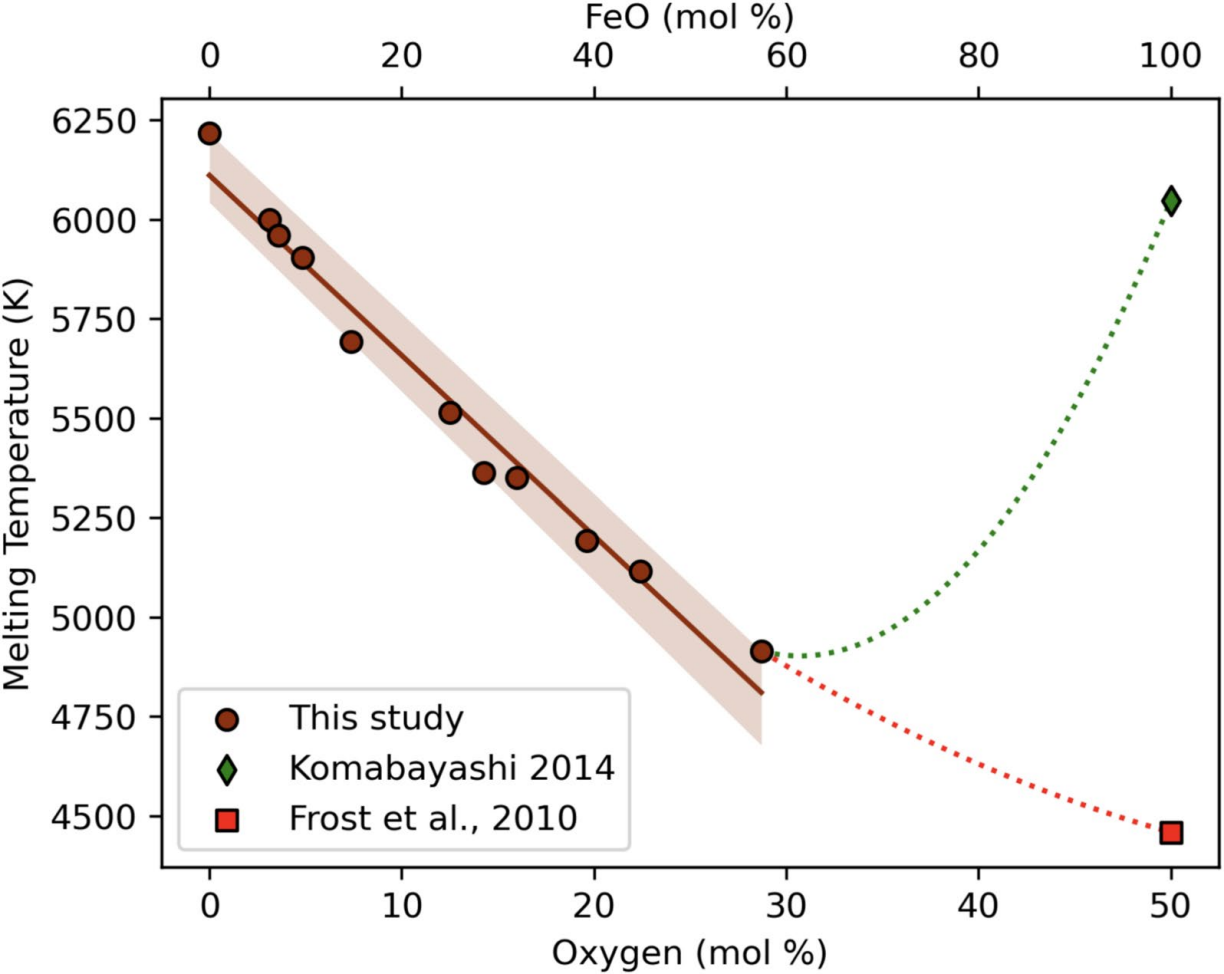
Melting temperatures calculated from two-phase coexistence simulations at 330 GPa (brown circles). Simulations of varying O fraction were performed, from 0 to 28.7 mol % (mol % O is shown on lower axis and mol % FeO on the upper axis). Results on the melting temperature of FeO from Komabayashi^26^ (green diamond) and Frost et al.^30^ (red square) are 6047 K and 4459 K, respectively.

The compositional fluctuation mechanism investigated in this paper depends primarily on the behaviour of the melting temperature and the probability of finding sub-volumes where the O concentration deviates significantly from the average. The melting temperature of $\hbox {Fe}_{1-x}\hbox {O}_{x}$ is calculated via two-phase coexistence simulations (see Ref.^19^ for details) at 330 GPa for *x* = 0–0.287 and results are presented in Fig. 1). As expected^19^, the melting temperature decreases monotonically with increasing O concentration within the range explored here. At *x* = 0.0 $T_{\textrm{m}}$ = 6215 K and $T_{\textrm{m}}$ decreases approximately linearly to *x* = 0.287 $T_{\textrm{m}}$ = 4915 K. For the FeO end-member (pure FeO), we show two previous estimates^26,30^ (square and diamond, Fig. 1). The two estimates differ by over 1500 K; however, what is important for this study is that the benefit for nucleation derived from compositional fluctuations relies on a large difference between the melting temperature of the bulk and the end-member compositions. Therefore, we expect that compositional fluctuations producing O-enriched regions will only reduce the nucleation waiting time $\tau _w$ when considering the high value^26^ of $T_{\textrm{m}}^{\textrm{FeO}}=6000$ K and so this is what we focus on below. We also assume that the eutectic point of the Fe-FeO system is at $\approx$ 30% O concentration, in line with previous estimates^26^.

**Fig. 2:**
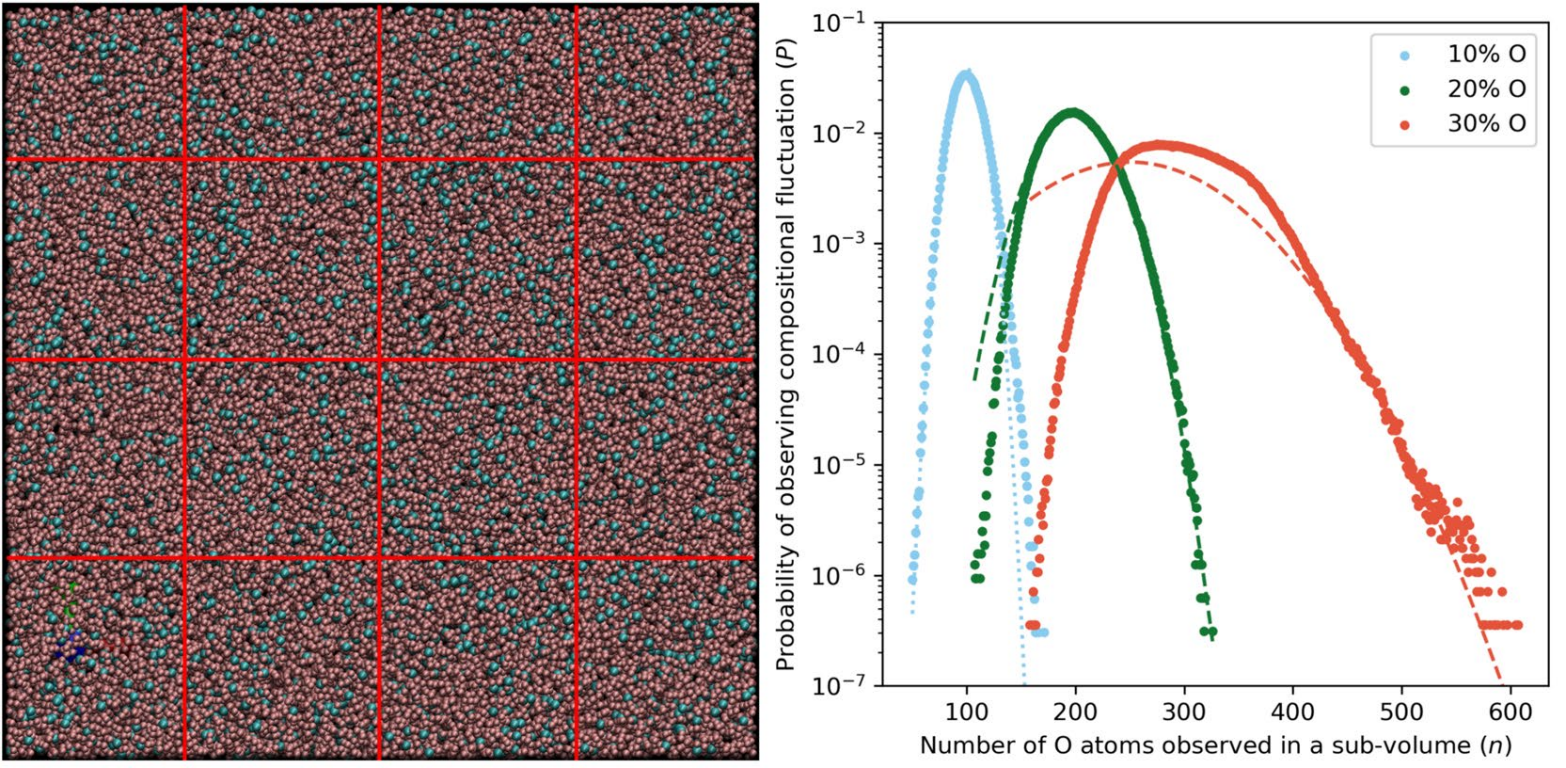
Left: A snapshot of a molecular dynamics simulation of Fe_0.9_O_0.1_ with 57,600 Fe (pink) and 6400 O (blue) atoms, at performed $P \approx 330$ GPa and $T = 5300$ K. Red lines indicate a $4\times 4\times 4$ fractioning of the simulation box in sub-volumes, each including 900 Fe and 100 O atoms. Right: Probability distributions for Fe systems with average compositions of 10, 20 and 30 mol.% O (blue, green and orange points, respectively). The probability is that of observing a simulation sub-volume (1/64 of the total volume) with *n* oxygen atoms, given a sub-volume contains 1000 atoms and 100 of these are O atoms on average for the 10 mol.% O case. A normal distribution is fit to underestimate the most rare low O concentration observations for the 10 % case (dotted line) and to underestimate the highest concentration observations in the 20 and 30 % cases (dashed lines).

Classical molecular dynamic simulations of $\hbox {Fe}_{1-x}\hbox {O}_{x}$ at 330 GPa and 5300 K are used to observe local fluctuations in composition in order to constrain the probability distribution *P*. These simulations of 64,000 atoms evolved for 237 ns, far larger and longer than our previous studies such that sufficient statistics on compositional fluctuations are gathered. At this temperature $\delta T<$ 300 K for all compositions tested, meaning that $\tau _w$ is extremely large and these simulations are not expected to spontaneously freeze. Every 1 ps a snapshot of atom positions is taken and sub-divided into 64 equal volumes. The composition of each is recorded to give a probability distribution of observing compositional variations from the mean (Fig. 2). Larger sub-volumes would result in fewer observations overall and poorer statistics of fluctuation probability distributions whereas smaller observed volumes would not capture the tails of the probability distribution. The random diffusion of atoms throughout the liquid system means that often O atoms will diffuse out of one sub-volume and into another, being replaced by an Fe atom and changing the composition of both sub-volumes. The composition of the bulk system is the most commonly observed composition. Heavily O enriched compositions are more likely to occur compared to equivalently O depleted compositions. For example, volumes containing 140 O atoms (40 more than average) represent $1\times 10^{-4}$ of observations whereas volumes containing 60 O atoms (40 less than average) represent $1\times 10^{-5}$ of observations. We shall return to this observation later.

Because of the limited time scale of the simulations the tails of the distribution, which are most relevant to inner core nucleation, are inaccessible. To mitigate this, we fit to fluctuations observed in our simulations by prioritising the rarest events at the expense of reproducing the frequency of the average composition, estimating the frequency of the rare events conservatively to avoid overestimating the effect of compositional fluctuations. We therefore fit the normal distribution given by Eq. (6) to the probability data in Fig. 2 to obtain $\sigma$ such that the least frequently observed, low O concentration sub-volumes are well represented for the 10 mol.% case. This approach means that the average composition of a system (which does not feature in our analysis) is slightly overestimated whilst rare low concentrations are, at worst, underestimated. The same approach is taken for high O fluctuations (20 and 30 mol.% cases), where the fit prioritises fluctuations towards FeO at the expense of overestimating the average composition. Ideally, extremely large calculations (> 1$\times 10^6$ atoms) would be employed to directly sample the tails of these distributions although our previous study^19^ compared nucleation in 7000 and 44,000 atom systems at core conditions and found that whilst smaller systems will sample fewer rare events they will accurately sample the events which they do produce.

To calculate the relevant size of a fluctuation that could trigger freezing and determine whether the low probability of pure regions occurring is offset by the shorter time needed to observe nucleation within them (lower $\tau _w$ due to locally increased $T_{\textrm{m}}$) we require information on the nucleation behaviour of the Fe and $\hbox {Fe}_{1-x}\hbox {O}_{x}$ systems, which we take from our previous studies^19,20^. Davies et al.^19^ conducted MD simulations at large enough $\delta T$ to observe freezing within the duration of a simulation, and fit $\tau _w$ in Eq. (5) with $\gamma$ and $I_0$ as free parameters, taking $h_f$ and $h_c$ from previous determinations of free energy in these systems^31^. Wilson et al.^20^ simulated pure iron at smaller $\delta T$ and used the observed distribution of small nuclei to extrapolate to $r_c$ using Eq. (2) with $\gamma$ and $g^{sl}$ as free parameters and calculating $\tau _0$ directly from simulations. The parameters used to calculate $\tau _w$ and $r_c$ here are taken from Ref.^19^, where $I_0^{Fe} = 7.1\times 10^{-49}$ s^−1^ m^−3^, $I_0^{FeO} = 7.9\times 10^{-46}$ s^−1^ m^−3^, $h_f = 0.98 \times 10^{10}$ J m^−3^, $h_c = 7.05\times 10^{-5}$, $\gamma ^{Fe} = 1.08$ and $\gamma ^{FeO} = 1.02$ J m^−2^. We assume that the statistics generated from the single pressure-temperature point in Fig. 2 adequately represents the statistics from the simulations spanning a broader *PT* range that were used to obtain these quantities.

**Fig. 3:**
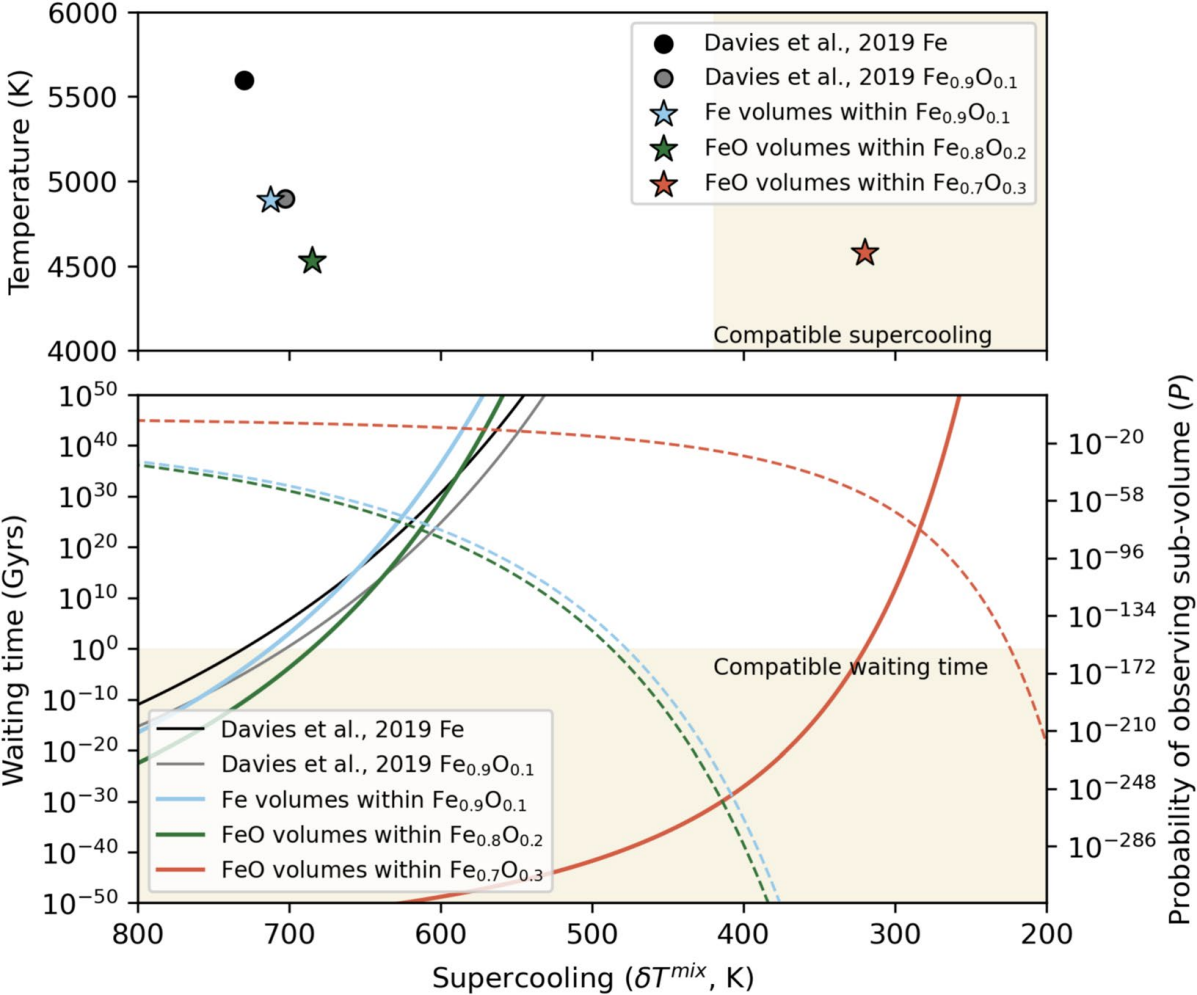
Upper panel: Supercooling and temperature required to nucleate the inner core (given 1 Gyrs of waiting time and a supercooled region in the core with a radius of 1221 km) from iron alloys with (stars) and without (circles) the effect of compositional fluctuations. Lower panel: Time to nucleate the Earth’s solid inner core vs supercooling. Pure Fe (black solid line^19^) and Fe_0.9_O_0.1_ (grey solid line^19^) nucleate at similar supercooling but have significantly differing $T_m$. Nucleation within pure Fe fluctuations of an Fe_0.9_O_0.1_ (blue solid line) requires a *T* intermediate to each isolated system but a smaller $\delta T$. Nucleation within FeO fluctuations of 20 and 30 mol % O systems (green and orange solid lines) requires $\delta T$ = 685 K and 320 K, respectively. The probability of finding a sub region of size $r_c$ is shown for pure iron volumes with Fe_0.9_O_0.1_ (blue dashed line) and FeO sub regions within Fe_0.8_O_0.2_ and Fe_0.7_O_0.3_ (green and orange dashed lines).

Figure 3 shows the waiting times $\tau _w$ and probabilities *P* for bulk concentrations with 10%, 20% and 30% O. A plausible upper limit on the $\tau _w$ that was available to the supercooled region of Earth’s core, $\tau _{IC}$ = 1 Gyr, is estimated assuming that the entire volume of the present-day inner core was supercooled for 1 Gyr^18,19,22^. The value of $\delta T$ required for a critical event to be observed within this time is then the minimum $\delta T$ that could trigger inner core nucleation for a given system. Geophysical constraints on $\delta T$ can be obtained by comparing a range of melting curves and adiabatic core temperature profiles, which gives $\delta T\lesssim$ 420 K (light green area, Fig. 3). For reference we show previous values^19^ of the $\delta T$ required to match $\tau _{IC}$ for pure Fe and Fe_0.9_O_0.1_ (black and grey lines, respectively) without considering compositional fluctuations, which are too large to match the $\delta T$ inferred from geophysical observations.

Figure 3 shows that the probability of finding an O-free volume of size $v_c$ in a system with bulk composition $c=0.1$ (blue dashed line) decreases strongly with increasing mixture supercooling $\delta T^{\textrm{mix}}$, falling below $10^{-200}$ at the largest geophysically acceptable supercooling of 400 K. At this mixture supercooling, the supercooling of pure Fe sub-volume is $\delta T^{\textrm{Fe}}\approx 1000$ K, but the reduction in $\tau$ in the same sub-volume is negated by the small *P* and the net effect on the overall $\tau _w$ is much too large to match $\tau _{IC}$ for Earth’s inner core. The $\delta T^{\textrm{mix}}$ of the bulk system required to match $\tau _w$ is around 700 K, far larger than geophysically compatible values, and so the fluctuation mechanism provides no benefit compared to nucleating from the bulk composition. We do not expect this result to change with increasing bulk O concentration as this will only further reduce the probability of observing O-free regions. We therefore conclude that compositional fluctuations producing O-depleted regions do not aid nucleation in the systems considered here.

Figure 3 also shows the probability of finding a region of pure FeO of volume $v_c$ in systems with bulk composition 0.2 and 0.3 (green and orange lines). As discussed above we assume an FeO melting temperature $T_{\textrm{m}}^{\textrm{FeO}}= 6200$ K. As for the O-depleted case, we fit the distribution in Eq. (6) to the data at high O concentrations in Fig. 2, in order to represent that part of the distribution as well as possible. For a 10% bulk oxygen concentration the probability of a fluctuation bringing the local concentration to 50% O is far too low, but for higher concentrations this probability starts to be relevant. For the 20% case, the combination of higher local supercooling and low probability of finding a region the size of the critical radius with 50% composition brings to the same conclusions found above for the oxygen depleted case: the supercooling required to match $\tau _{IC}$ is still too large (i.e. nearly 700 K), providing no benefit to nucleation. However, for the 30% oxygen bulk composition the further reduction in melting temperature (which increases the local effective undercooling) and the increased probability of a composition fluctuation taking the critical region to 50% oxygen composition mean that the supercooling required is only just above 300 K. This is well within the required geophysical constraints, though the bulk oxygen composition is probably far too large to represent a viable composition for the Earth’s core^25,27^.

## Discussion

We have shown that significant atomic-scale variations in chemical concentration arise in supercooled $\hbox {Fe}_{1-x}\hbox {O}_{x}$ liquids at the pressure and temperature conditions of Earth’s core. Regions of nearly pure Fe are favourable for homogeneous nucleation because the supercooling is large (the melting point is locally elevated); however, the probability of producing a region large enough to facilitate nucleation of the mixture is so low as to negate the favourable supercooling and there is no overall benefit for nucleation. Higher bulk O concentrations than we have considered would only further lower the probability, while lower bulk O concentrations would limit the melting point depression that helps to facilitate nucleation. Therefore our findings do not indicate that compositional fluctuations producing O-depleted regions are a viable means to reduce the requirements for nucleating Earth’s inner core.

We have also found a significant asymmetry about the average composition in the probability distribution for producing regions large enough to facilitate nucleation: O-enriched regions are much more common than O-depleted regions. This phenomenon could be related to liquid structure around O atoms modifying the transport properties within these regions^32^ but these details require additional attention beyond the scope of the present study. Regions of nearly pure FeO can reduce the overall supercooling required for nucleation assuming an FeO melting temperature comparable to that of pure Fe^26^ if the bulk O concentration is high enough ($\gtrsim 20$ mol%). Our results show that with a bulk O concentration of 30 mol%, a geophysically acceptable supercooling of $\delta T\approx 300$ K can match the waiting time that is available to nucleate Earth’s inner core. However, such high O concentrations are incompatible with constraints from seismology, core formation studies, and cosmochemistry, which limit the core’s O content to^25,27,33^ $\lesssim 20$ mol%. For this range of core O concentrations, the fluctuation mechanism reduces the supercooling of the bulk system by a maximum of $\sim 50$ K and hence the mixture supercooling remains around 700 K, which is far too high to be compatible with geophysical constraints.

Our analysis of compositional fluctuations in MD simulations relies on a number of assumptions and approximations. The normal distribution used to fit the simulation data tends to underestimate the probability at high and low O concentrations. Ideally another distribution would be used, but we have not succeeded in improving on the fits presented in Fig. 2. The range of data used for the fitting also has an effect on the value of the parameter $\sigma$ and, as a result, it is responsible for error on the estimate of the supercooling needed for Eq. (5) to match the inner core waiting time. We estimate this error to be of the order of $\approx 50$ K. Finally, the melting point of FeO is a crucial parameter. We have used the value of 6200 K in Fig. 1; using the lower value of $\sim 4400$ K would completely remove any benefit from compositional fluctuations due to O-enriched regions. Overall, the results presented here do not indicate that any of these issues affect our overall conclusion that compositional fluctuations alone are not able to explain the nucleation of Earth’s inner core.

Compositional fluctuations in the iron alloys offer a route to reducing the supercooling required to nucleate Earth’s solid inner core. However, the reductions we have found are not enough to bring the predicted supercooling into the geophysically acceptable range for a plausible core composition. Furthermore, other constraints on the maximum $\delta T$ of the core, including the long term generation of the geomagnetic field and the presence of heterogeneous inner core structure, only allow for $\delta T\le$ 100 K (see Ref.^17^ for a review). In this case, the simple binary compositions explored here and in previous studies seem unable to explain inner core formation. Exploration of ternary systems, which are required for a complete description of the core’s seismic properties^34,35^, are the obvious next step in the pursuit of a valid nucleation mechanism and compositional fluctuations could be crucial in these systems. For example, Fe-O-C systems might benefit from low bulk $T_m$ and Fe-C sub-volumes with high $T_m$ and elevated $I(r_c)$. In this case, enriching a sub-volume in C may come with the added benefit of enrichment being more probable than equivalent depletion (if C behaves similarly to O in this regard), as shown in the results presented here. Indeed, it is interesting to note the high sensitivity of our results to the actual bulk composition, which may suggest that the mechanism described here could work for a different system. For this to be possible, compositional fluctuations in other system would need to behave similarly.

## Methods

### Molecular dynamic simulations

Our classical molecular dynamic (CMD) simulations follow the approach of Davies et al.^25^, using the same embedded atom model (EAM) as the previous study to represent Fe and O atoms. This interatomic potential has previously been validated against ab initio calculations^19,36^ and allows the efficient modelling of large numbers of atoms over long durations, which is not possible with ab initio calculations. EAMs define the energy of a system (*E*) as the sum of energies arising from pairwise interactions between each atom (*i*) and its neighbours (*j*). In a system composed of Fe and O atoms, three unique interactions exist

8 $$\begin{aligned} E = \sum _{i = 1}^{N_{Fe}} E_{i}^{Fe} + \sum _{i = 1}^{N_{O}}E_{i}^{O} +\sum _{i = 1}^{N_{Fe O}}E_{i}^{Fe O}. \end{aligned}$$

Each interaction is comprised of an embedded (*F*) and a repulsive (*Q*) term

9 $$\begin{aligned} E_{i}^{Fe} = Q_{i}^{Fe} + F^{Fe}(\rho _{i}^{Fe}) = \sum _{j=1, j \ne i}^{N_{Fe}} \epsilon ^{Fe} \left( a^{Fe}/r_{ij} \right) ^{n^{Fe}} - \epsilon ^{Fe} C^{Fe} \sqrt{\rho _{i}^{Fe}}, \end{aligned}$$

10 $$\begin{aligned} E_{i}^{O} = Q_{i}^{O} + F^{O}(\rho _{i}^{O}) = \sum _{j=1, j \ne i}^{N_{O}} \epsilon ^{O} \left( a^{O}/r_{ij} \right) ^{n^{O}} - \epsilon ^{O} C^O \sqrt{\rho _{i}^{O}}, \end{aligned}$$

11 $$\begin{aligned} E_{i}^{FeO} = Q_{i}^{FeO} = \frac{1}{2} \sum _{i=1}^{N_{Fe}} \sum _{j=1, i \ne j}^{N_{O}} \epsilon ^{Fe O} \left( a^{Fe O}/r_{ij} \right) ^{n^{Fe O}}, \end{aligned}$$

where *a*, *n*, $\epsilon$, and *C* are the free parameters of each interaction, fit to ab initio data, and $r_{ij}$ and $\rho _{ij}$ are the length separation and electron density between pairs of atoms. Electron density for each interaction is defined as

12 $$\begin{aligned} \rho_{i_{Fe}} = \sum{_{{j_{Fe}} \neq i_{Fe}}} {\left( a^{Fe} / r_{{i_{Fe}}j_{Fe}}\right)^{m^{Fe}}} + \sum{_{{j_{O}}}} {\left( a^{FeO} / r_{{i_{Fe}}j_{O}}\right)^{m^{FeO}}}\end{aligned}$$

13 $$\begin{aligned} \rho_{i_{O}} = \sum{_{{j_{O}} \neq i_{O}}} {\left( a^{O} / r_{{i_{O}}j_{O}}\right)^{m^{O}}} + \sum{_{{j_{Fe}}}} {\left( a^{FeO} / r_{{i_{O}}j_{Fe}}\right)^{m^{FeO}}}\end{aligned}$$

CMD simulations have periodic boundary conditions, are conducted in the NVT ensemble and contain 64000 atoms with 6400, 12800, 16000 and 19200 of these being O atoms for 10, 20, and 30 mol. % O simulations, respectively. The volume of simulations is tuned to give a pressure of $\sim$ 330 GPa at 5300 K. Simulations are melted at a temperature of 10,000 K for 1 ps of simulation time to achieve a random, fully liquid configuration before cooling to the target temperature of 5300 K. Simulations are evolved for 237 ns in a single trajectory with a timestep of 1 fs and the composition of each $\frac{1}{64}$^th^ (4$\times$4$\times$4 grid) is recorded every 1000 timesteps.

## Data Availability

The datasets generated and analysed during the current study are available in the following Zenodo repository: https://doi.org/10.5281/zenodo.15016846
