# Correction: The effect of compositional fluctuations in a liquid Fe–O alloy on the nucleation of Earth’s inner core

**DOI:** 10.1038/s41598-025-20530-8

**Published:** 2025-09-26

**Authors:** Alfred J. Wilson, Monica Pozzo, Christopher J. Davies, Andrew M. Walker, Dario Alfè

**Affiliations:** 1 School of Earth and Environment, University of Leeds, Leeds, LS2 9JT UK; 2 Faculty of Technological and Innovation Sciences, Universitas Mercatorum, 00186 Rome, Italy; 3 Department of Earth Sciences, University College London, London, WC1E 6BT UK; 4 Institute for Materials Discovery, UCL East, London, E20 2A UK; 5 London Centre for Nanotechnology, Thomas Young Centre, University College London, London, UK; 6 Department of Earth Sciences, University of Oxford, Oxford, OX1 2JD UK; 7 London Centre for Nanotechnology, University College London, London, WC1H 0AH UK; 8 Dipartimento Di Fisica “Ettore Pancini”, Università Di Napoli Federico II, 80126 Naples, Italy

Correction to: *Scientific Reports* 10.1038/s41598-025-07258-1, published online 1 July 2025

The original version of this Article contained errors.

Equation 12 was incorrectly given as:

$$\begin{array}{*{20}c} {\rho_{i}^{Fe} = \mathop \sum \limits_{j = 1,j \ne i}^{{N_{Fe} }} \left( {a^{Fe} /r_{ij} } \right)^{{m^{Fe} }} + \rho_{i}^{Fe} } \\ \end{array}$$

The correct Eq. 12 appears below.

12 $$\rho_{{i_{Fe} }} = \mathop \sum \limits_{{j_{Fe} \ne i_{Fe} }} \left( {a^{Fe} /r_{{i_{Fe} j_{Fe} }} } \right)^{{m^{Fe} }} + \mathop \sum \limits_{{j_{O} }} \left( {a^{FeO} /r_{{i_{Fe} j_{O} }} } \right)^{{m^{FeO} }}$$

In addition, Eq. 13 was incorrectly given as:

$$\begin{array}{*{20}c} {\rho_{i}^{O} = \mathop \sum \limits_{j = 1,j \ne i}^{{N_{O} }} \left( {a^{O} /r_{ij} } \right)^{{m^{O} }} + \rho_{i}^{FeO} } \\ \end{array}$$

The correct Eq. 13 appears below.

13 $$\rho_{{i_{O} }} = \mathop \sum \limits_{{j_{O} \ne i_{O} }} \left( {a^{O} /r_{{i_{O} j_{O} }} } \right)^{{ m^{O} }} + \mathop \sum \limits_{{j_{Fe} }} \left( {a^{FeO} /r_{{i_{O} j_{Fe} }} } \right)^{{m^{FeO} }}$$

Finally, Eq. 14 has been removed from the Article.

$$\begin{array}{*{20}c} {\rho_{i}^{FeO} = \mathop \sum \limits_{j = 1,j \ne i}^{{N_{O} }} \left( {a^{FeO} /r_{ij} } \right)^{{m^{FeO} }} } \\ \end{array}$$

The original Article has been corrected.

